# Study on the Structural Characteristics and Foaming Properties of Ovalbumin—Citrus Pectin Conjugates Prepared by the Maillard Reaction

**DOI:** 10.3390/foods13223542

**Published:** 2024-11-06

**Authors:** Shanshan Zhang, Yibo Liu, Wenhui Wu

**Affiliations:** 1 Department of Marine Biopharmacology, College of Food Science and Technology, Shanghai Ocean University, Shanghai 201306, China; zss13139939001@163.com; 2 School of Food Science and Technology, Shihezi University, Shihezi 832003, China; lyb14235@163.com; 3 Marine Biomedical Science and Technology Innovation Platform of Lin-Gang Special Area, Shanghai 201306, China

**Keywords:** ovalbumin, citrus pectin, Maillard reaction, structure, foaming properties

## Abstract

This study explored the structural features and foaming properties of ovalbumin (OVA) and its glycosylated conjugates with citrus pectin (CP) formed through the Maillard reaction. The results demonstrated that OVA and CP were successfully conjugated, with the degree of grafting increasing to 43.83% by day 5 of the reaction. SDS-PAGE analysis confirmed the formation of high-molecular-weight conjugates. Fourier-transform infrared (FT-IR) and fluorescence spectroscopy further revealed alterations in the secondary and tertiary structures of OVA, including an enhanced β-sheet content, a reduced β-turn content, and the depletion of tryptophan residues. Moreover, the surface hydrophobicity of the OVA–CP conjugates significantly increased, enhancing foaming properties. Furthermore, the analysis of foaming properties exhibited that the Maillard reaction improved the foaming capacity of OVA to 66.22% and foaming stability to 81.49%. These findings highlight the potential of glycosylation via the Maillard reaction to significantly improve the foaming properties of OVA, positioning it as a promising novel foaming agent.

## 1. Introduction

Foams are extensively used in the food industry, especially in products like protein drinks, dairy foams, and baked goods [1]. The stability of food foams is crucial for maintaining texture, appearance, and consistency, depending heavily on factors such as liquid viscosity, interfacial tension, and the properties of the foaming agents [2,3]. To stabilize foams, foaming agents or surfactants are employed to reduce interfacial tension and prevent bubble coalescence. Among natural foaming agents, proteins are particularly important due to their amphiphilic structure and surface activity, which enhance foam stability [4]. However, many natural proteins face limitations in generating and maintaining foam, especially those with higher molecular weights or compact structures that may not expand effectively into the interfacial layer of foams, resulting in unstable films and compromised foaming performance [5]. To address these shortcomings, various modification techniques have been explored to improve protein foaming properties and stabilize food foams [6].

Ovalbumin (OVA), the primary protein in egg white, constitutes approximately 54% of its total protein content [7]. Known for its excellent gelling and emulsifying abilities, OVA is commonly used in food applications, including dairy products, baked goods, and beverages [8]. However, the rigid tertiary structure of OVA, stabilized by disulfide bonds and hydrophobic interactions, limits its ability to unfold and diffuse rapidly at interfaces, hindering the formation of stable films around bubbles and reducing foam stability [9]. Although proteins with higher surface hydrophobicity can improve foam formation by lowering surface tension, the natural hydrophobicity of OVA is suboptimal, delaying its interfacial diffusion [10]. Additionally, OVA tends to aggregate during foaming, further compromising foam stability. To overcome these limitations, researchers have investigated various modification methods, such as heat treatment [11], pH adjustment [12], and enzymatic processes [13], to improve the foaming properties of OVA.

Among these modification techniques, glycosylation, particularly non-enzymatic glycosylation through the Maillard reaction, has emerged as a promising approach for improving protein functionality [14]. This method is safe, requires no chemical additives, and utilizes natural food ingredients, making it highly applicable in the food industry [14,15]. Studies have shown that glycosylation significantly enhances the foaming properties of proteins. For example, soybean protein isolate hydrolysates conjugated with xanthan gum through the Maillard reaction demonstrated increased interfacial adsorption and reduced surface tension, leading to improved foaming performance [16]. Similarly, the glycosylation of gluten protein with fructose increased its foaming capacity nearly threefold [17]. In addition, the foaming capacity of soybean isolate protein–peach gum conjugates formed through a wet Maillard reaction was significantly improved [18].

Citrus pectin (CP), a natural polysaccharide characterized by high water solubility and surface activity, is widely used in the food industry [19]. Rich in galacturonic acid, CP exhibits high reactivity in the Maillard reaction, making it particularly suitable for glycosylation to enhance protein functional properties [20]. Additionally, the branched structure of CP can synergize with proteins, forming a dense and stable interfacial layer around bubbles, thus improving foam stability. Despite these advances, the glycosylation of OVA with CP to improve its foaming properties remains unexplored.

This study aimed to prepare OVA–CP conjugates through the Maillard reaction and to investigate their structural characteristics and foaming properties. Structural changes in OVA, both before and after the Maillard reaction, were analyzed using SDS-PAGE, Fourier-transform infrared spectroscopy, fluorescence spectroscopy, and surface hydrophobicity measurements. Additionally, the foaming properties of OVA and OVA–CP conjugates were evaluated. The objective of this study is to improve the foaming properties and foaming stability of OVA through glycosylation and to reveal the correlation between OVA structure and foaming properties. These findings will address the limitations of OVA and expand its potential applications in the food industry.

## 2. Materials and Methods

### 2.1. Materials

Ovalbumin and citrus pectin were obtained from Sigma-Aldrich (St. Louis, MO, USA). O-phthalaldehyde, Coomassie Brilliant blue G-250, and 8-amino-1-naphthalenesulfonic acid (ANS) were supplied from China National Pharmaceutical Group Chemical Reagent Co., Ltd. (Shanghai, China).

### 2.2. Preparation of OVA–CP Conjugates

A mixture of ovalbumin and citrus pectin in a weight ratio of 1:2 was dissolved in 10 mM phosphate buffer at pH 7.0. The solution was frozen at −80 °C for 24 h, then freeze-dried at −60 to −70 °C under a vacuum pressure of 0 Pa for 48 h, and subsequently ground into a powder. The samples were subsequently incubated in a controlled temperature and humidity chamber at 60 °C and 79% relative humidity for 0 to 7 days [8]. After the reaction, the obtained OVA–CP conjugates were stored in a refrigerator at 4 °C until further experiments. The samples obtained after 0, 1, 2, 3, 4, 5, 6, and 7 days of reaction were designated as Mixture, OVA–CP–1d, OVA–CP–2d, OVA–CP–3d, OVA–CP–4d, OVA–CP–5d, OVA–CP–6d, and OVA–CP–7d, respectively.

### 2.3. Evaluation of the Degree of Grafting and Browning

The free amino content of the glycosylation products was determined using the o-phthalaldehyde (OPA) method [21]. A total of 4 mL of the OPA reagent was added to 200 μL of a 4 mg/mL sample solution. The mixture was mixed thoroughly and incubated at 35 °C for 2 min, after which the absorbance was promptly measured at 340 nm with a UV-2700 spectrophotometer (Shimadzu Corp., Kyoto, Japan). The degree of grafting was measured using Equation (1):

(1) $$\mathrm{Degree}\;\mathrm{of}\;\mathrm{grafting}\;(\%)=\frac{A-A_{d}}{A}\times 100$$

where *A* represents the absorbance of the solution at 0 d and *A_d_* represents the absorbance at d days.

The degree of browning was evaluated by measuring the absorbance at 420 nm (A_420_).

### 2.4. SDS-PAGE

SDS-PAGE analysis was executed using gel plates, with the stacking gel prepared from a 5% acrylamide solution in 1.0 M Tris-HCl buffer and the separating gel prepared from a 12% acrylamide solution in 2.0 M Tris-HCl buffer. The migration process was conducted at 80 V in the stacking gel and at 120 V in the separating gel, utilizing a buffer containing 25 mM Tris, 192 mM glycine, and 0.1% SDS, pH 8.3. The samples were dissolved or diluted to a protein concentration of 2 mg/mL, mixed with loading buffer, and boiled for 5 min before loading 10 μL into each lane of the gel. Standard proteins ranging from 10 to 250 kDa were used as markers. After electrophoresis, the gel was stained with 0.2% Coomassie Brilliant blue R-250 for 30 min. The gel was then immersed in a solution of 10% acetic acid and 10% ethanol for destaining. The other gel was also stained with 0.5% periodic acid–Schiff (PAS) reagent [22].

### 2.5. Fourier Transform Infrared Spectroscopy (FT-IR)

The freeze-dried samples were ground with KBr at a ratio of 1:100 and pressed into uniform, transparent tablets using a tablet press. The infrared spectra of OVA and OVA–CP conjugates were recorded by an FT-IR spectrometer (VERTEX 70v, Bruker Ltd., Berlin, Germany) over a wavelength range of 400–4000 cm^−1^ with a resolution of 4 cm^−1^. The analysis of protein secondary structure was performed using Peakfit V4.2 software.

### 2.6. Intrinsic Fluorescence Spectroscopy

Intrinsic fluorescence emission spectra were recorded using a fluorescence spectrophotometer (F-7000, Hitachi Ltd., Tokyo, Japan). Samples were dissolved in a 10 mM phosphate buffer at pH 7.0 with a protein concentration of 0.1 mg/mL. The fluorescence spectrophotometer was set to a slit width of 5 nm, with an excitation wavelength of 280 nm and an emission wavelength range from 300 to 450 nm, scanning at a speed of 700 nm/min [23]. The phosphate buffer served as a blank for the measurements.

### 2.7. Determination of Surface Hydrophobicity

Surface hydrophobicity was measured using the ANS fluorescence probe method [24]. The samples were prepared in 10 mM phosphate buffer at concentrations of 0.05, 0.1, 0.2, 0.3, and 0.4 mg/mL. A total of 4 mL of each protein solution was mixed with 40 μL of an 8 mmol/L ANS solution and allowed to react in the dark for 15 min. The fluorescence intensity was measured with an excitation wavelength of 390 nm, an emission wavelength of 470 nm, and a slit width of 5 nm. The surface hydrophobicity was calculated by plotting fluorescence intensity against protein concentration, with the slope of the fitted line representing the surface hydrophobicity index of the sample.

### 2.8. Determination of Free and Total Sulfhydryl Content

The total sulfhydryl content was determined using Ellman’s reagent with DTNB, following the method described by Ma et al. [25]. The free and total sulfhydryl content were calculated using Equation (2):

(2) $$-\mathrm{SH}\;(\mu \mathrm{mol}/g)=\frac{75.53\times A_{412}\times D}{C}$$

where *A*_412_ represents the absorbance of the samples, *D* is the dilution factor (5), and *C* is the protein concentration of the samples (mg/mL).

### 2.9. Measurement of Particle Size and Zeta Potential

The samples were dissolved in 10 mM phosphate buffer (pH 7.0) at a protein concentration of 1 mg/mL. The particle size and zeta potential of the samples were measured using a nanoparticle sizing instrument (ZS90, Malvern Instrument Ltd., Malvern, UK). Each sample was analyzed in triplicate.

### 2.10. Measurement of Solubility

A 1 mg/mL sample solution was dissolved in 10 mL of phosphate buffer (pH 7.0). The sample solution was centrifuged at 5000 r/min for 15 min using a centrifuge (SL1 Plus, Thermo Fisher Scientific Inc., Carlsbad, CA, USA). Solubility was determined by expressing the total protein content present in the supernatant as a percentage [26].

### 2.11. Measurement of Foaming Properties

The foaming properties of OVA and the OVA–CP conjugates were measured following the method described by Yang et al. [27]. A 30 mL solution of samples at a concentration of 15 mg/mL was sheared at 12,000 r/min in a 100 mL graduated cylinder for 2 min. The initial liquid volume (*V*_0_), total volume of the solution and foam after 2 min of stirring (*V*_1_), and the total volume after 30 min of standing (*V*₂) were measured. The foaming capacity and stability were determined by Equations (3) and (4):

(3) $$\mathrm{Foaming}\;\mathrm{capacity}\;(\%)=\frac{V_{1}-V_{0}}{V_{0}}\times 100$$

(4) $$\mathrm{Foaming}\;\mathrm{stability}\;(\%)=\frac{V_{2}}{V_{1}}\times 100$$

### 2.12. Foam Microstructure

The foam samples, as described in Section 2.11, were observed 30 min after standing using an inverted fluorescence microscope (IX71, Olympus Cor., Tokyo, Japan) in optical mode, at a magnification of 40×.

### 2.13. Statistical Analysis

All experimental data are presented as means ± standard deviation from three replicates. Differences among means were deemed significant at *p* < 0.05 using Duncan’s test. Statistical analysis was conducted with SPSS 26.0 (SPSS Inc., Chicago, IL, USA).

## 3. Results

### 3.1. Analysis of the Degree of Maillard Reaction

**Figure 1:**
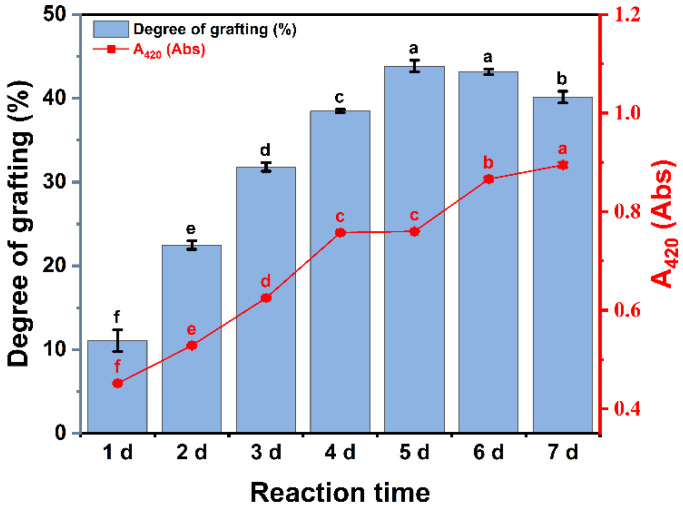
The degree of grafting and browning (A_420_) of OVA–CP conjugates. Different letters indicate significant differences (*p* < 0.05).

The degree of grafting measures the extent of covalent bonding between proteins and polysaccharides, while the degree of browning reflects the formation of Maillard reaction compounds [28]. The results for the degree of grafting and browning are illustrated in Figure 1. As the Maillard reaction progressed, the free amino groups of OVA reacted with the carbonyl groups of CP, leading to a gradual increase in the grafting degree of the OVA–CP conjugates, which reached a maximum of 43.83% by day 5. After this point, the grafting degree stabilized. The observed increase in the degree of grafting indicated a reduction in free amino group content. Prolonged reaction times resulted in fewer available carbonyl and amino groups, contributing to the slowing rate of the increase in grafting. This trend aligns with previous research, which reports a rapid increase in grafting during the early stages of the Maillard reaction, followed by stabilization over time [20]. Additionally, throughout the Maillard reaction process, browning values ranged between 0.3 and 0.9 Abs, gradually increasing with extended reaction time. This result aligns with the findings of Wang et al. [29]. The observed browning may result from protein denaturation and the exposure of free amino groups over extended reaction periods, facilitating glycosylation reactions and the formation of melanoidin-like compounds through aldol–amino interactions [30]. Consequently, this results in elevated browning values. These findings confirm that the Maillard reaction between OVA and CP induced covalent linking, forming chromophores and contributing to the observed increase in browning.

### 3.2. SDS-PAGE Analysis

**Figure 2:**
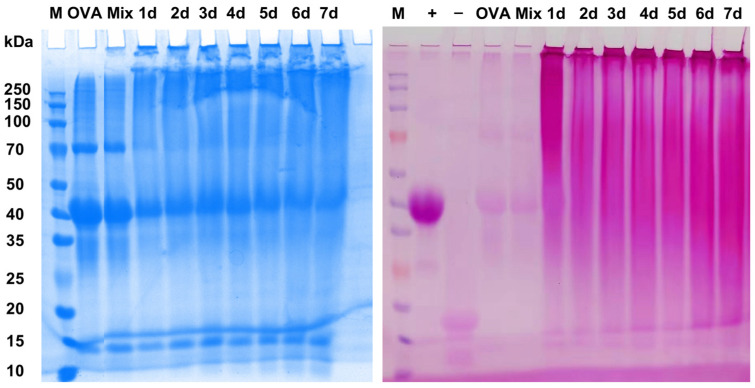
SDS-PAGE analysis of native OVA, Mixture, and OVA–CP conjugates stained by Coomassie blue and periodic acid–Schiff (PAS) reagent, respectively.

SDS-PAGE analysis was conducted to assess glycosylation modification, subunit composition, and molecular weight changes in the proteins. The SDS-PAGE results for OVA and the OVA–CP conjugates are presented in Figure 2. The molecular weight of OVA was approximately 45 kDa, consistent with previous studies [8]. After mixing with CP, the Mixture exhibited the same characteristic bands as OVA, indicating that the introduction of CP did not alter the protein structure. However, as the Maillard reaction progressed, the characteristic 45 kDa band of the OVA–CP conjugates gradually faded compared to natural OVA. Concurrently, a broad new band appeared within the molecular weight range of 70–200 kDa, likely due to covalent bonding between OVA and CP, which increased the molecular weight of the protein. The pink bands in Figure 2, stained using the periodic acid–Schiff (PAS) reagent, indicate glycoproteins. The intensity of these pink bands reflects the extent of OVA–CP conjugate formation, with deeper shades indicating higher levels of glycosylation. No pink bands were observed in the lanes corresponding to OVA and the mixture, confirming the absence of protein–polysaccharide conjugates in these samples. Additionally, the presence of high-molecular-weight proteins that remained in the stacking gel and did not enter the separating gel suggests that the OVA–CP conjugates formed high-molecular-weight polymers via covalent bonding, a phenomenon consistent with previous findings [31].

### 3.3. Fourier-Transform Infrared (FT-IR) Spectroscopy and Secondary Structure of Proteins Analysis

**Figure 3:**
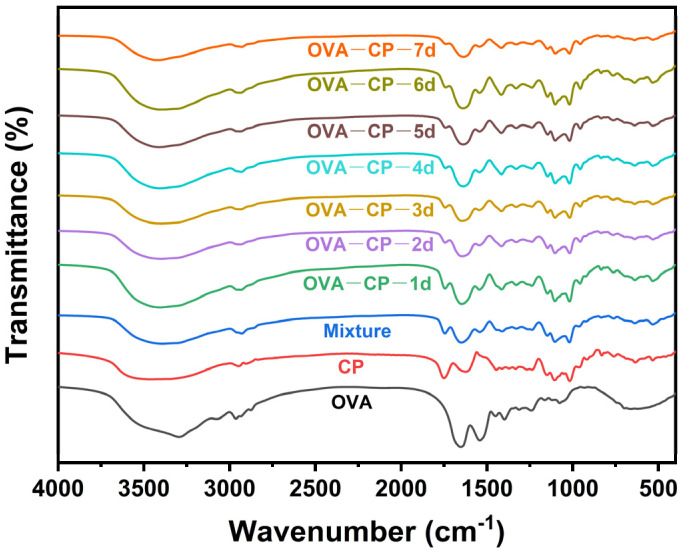
FT-IR spectroscopy of native OVA, Mixture, and OVA–CP conjugates.

The FT-IR spectra of OVA and the OVA–CP conjugates are shown in Figure 3. Compared to native OVA, the spectrum of the OVA–CP conjugates exhibited significant differences. After glycosylation modification, the OVA–CP conjugates showed an enhanced peak in the range of 3700–3200 cm^−1^, corresponding to the stretching of O–H bonds due to the hydroxyl groups introduced by CP [32]. The peaks at 1743 cm^−1^ and 1013 cm^−1^, characteristic peaks of CP [26], appeared with varying intensities in the OVA–CP conjugates, suggesting successful covalent bonding between CP and OVA. Additionally, the amide I (1700–1600 cm^−1^), amide II (1550–1500 cm^−1^), and amide III (1300–1200 cm^−1^) bands are characteristic spectra of OVA [8]. After the Maillard reaction with CP, these characteristic absorption peaks showed varying degrees of enhancement or reduction. Similar modifications to amide bands have been observed in previous studies on the Maillard reaction between soybean isolate proteins and polysaccharides like guar gum and carrageenan [33].

This structure primarily consists of α-helix, β-sheet, and β-turn conformations, while random coil regions lack a stable structure [34]. As shown in Table 1, OVA and all the OVA–CP conjugates contain four types of secondary structures. In native OVA, β-sheet and β-turn structures predominate, accounting for 35.33% and 40.34%, respectively. In the Mixture with CP, the β-turn content decreased to 33.03%, while the α-helix increased to 22.77%. After glycosylation modification, the OVA–CP conjugates exhibited a significant reduction in β-turn and an increase in β-sheet content. This structural change can be attributed to the covalent bonding between CP and OVA during the Maillard reaction, which alters the protein secondary structure. These findings show that the Maillard reaction substantially affects the secondary structure of OVA.

### 3.4. Intrinsic Fluorescence Spectroscopy Analysis

**Figure 4:**
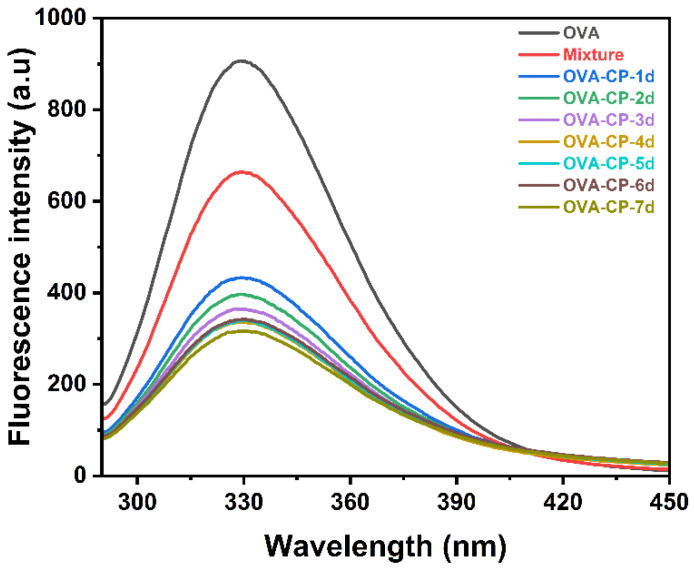
Intrinsic fluorescence spectroscopy of native OVA, Mixture, and OVA–CP conjugates.

Intrinsic fluorescence spectroscopy analysis of proteins can be used to monitor changes in the microenvironment surrounding tryptophan residues, providing insights into alterations in the tertiary structure of OVA. Figure 4 presents the intrinsic fluorescence spectra of the OVA–CP conjugates subjected to various glycosylation durations. When the excitation wavelength was set to 280 nm, all samples exhibited maximum fluorescence intensity at 330 nm. When CP was mixed with OVA, the fluorescence intensity of the Mixture considerably decreased, likely due to the chain structure of CP adhering to the surface of OVA, thereby shielding the tryptophan residues [22]. Following glycosylation, the fluorescence intensity of the OVA–CP conjugates was poorer than that of OVA, with a more pronounced reduction observed as the glycosylation treatment duration increased. The decrease in fluorescence intensity indicates that the tryptophan residues were situated in a more revealed, polar microenvironment [8]. The reduction in fluorescence is attributed to the glycosylation modifications, which likely induced changes in the tertiary structure by reducing the tryptophan content within the protein [35]. These findings are consistent with the results reported by Liu et al. [22], who observed a similar decrease in the fluorescence intensity of chickpea protein isolate at 320 nm after reacting with citrus pectin via the Maillard reaction. Conversely, the attachment of CP to OVA also appears to partially shield the tryptophan residues, thereby reducing the quantum yield of fluorescence.

### 3.5. Surface Hydrophobicity Analysis

**Figure 5:**
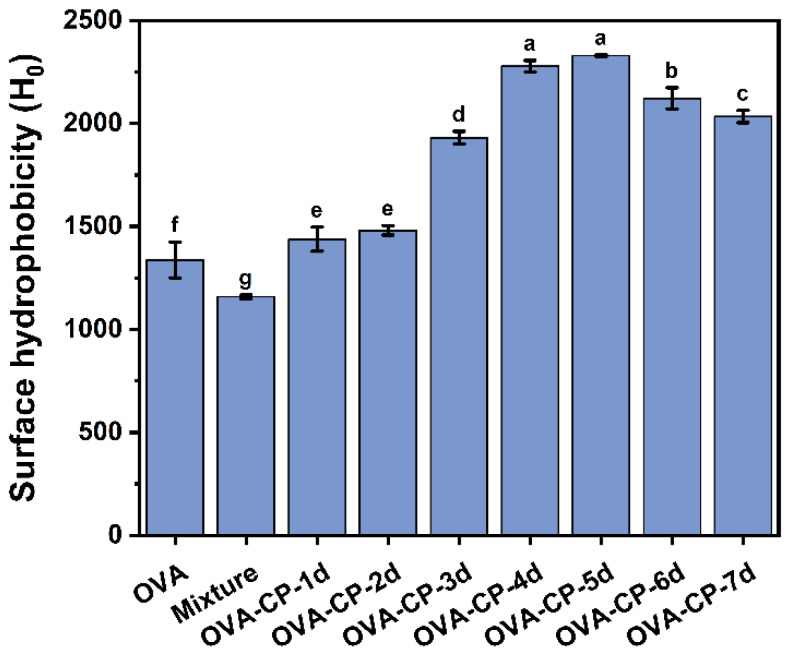
Surface hydrophobicity of native OVA, Mixture, and OVA–CP conjugates. Different letters indicate significant differences (*p* < 0.05).

Surface hydrophobicity is a critical parameter for evaluating the structural changes and functional characteristics of proteins, particularly in their interactions with protein solutions and air, which directly influence foaming properties. Surface hydrophobicity is closely related to the behavior of proteins in aqueous solutions and is typically associated with the extent of exposure of hydrophobic regions within the protein molecule [36]. Assessing surface hydrophobicity is essential for evaluating the structural changes in proteins induced by the Maillard reaction and their subsequent effects on surface adsorption behavior and functional properties. As glycosylation progressed, the surface hydrophobicity of the OVA–CP conjugates initially increased, reaching a maximum at day 5 (2329.67), before slightly decreasing (Figure 5). Native OVA exhibited relatively low surface hydrophobicity (1337.60), while the Mixture showed similar values to OVA. The rise in surface hydrophobicity with extended glycosylation indicates significant structural changes in OVA, potentially due to the exposure of hydrophobic groups as the protein structure loosens. This increase in surface hydrophobicity enhances the ability of protein to adsorb at the air/water interface, which could lead to improved foaming properties. However, after day 5 of glycosylation, the slight decrease in surface hydrophobicity may be due to excessive reactions, leading to protein aggregation or structural breakdown, causing some hydrophobic groups to be re-buried within the molecule. Alternatively, dense covalent glycosylation may prevent the effective exposure of hydrophobic regions. These findings align with the research of Nasrollahzadeh et al. [37], who reported an initial increase followed by a decrease in surface hydrophobicity during the glycosylation of bovine serum albumin, suggesting a gradual loosening of the protein structure. Overall, these results suggest that glycosylation modifications can significantly enhance the surface hydrophobicity of OVA, thereby improving its foaming properties. However, excessive glycosylation may induce further structural changes that could negatively impact functional properties.

### 3.6. Free and Total Sulfhydryl Content Analysis

**Figure 6:**
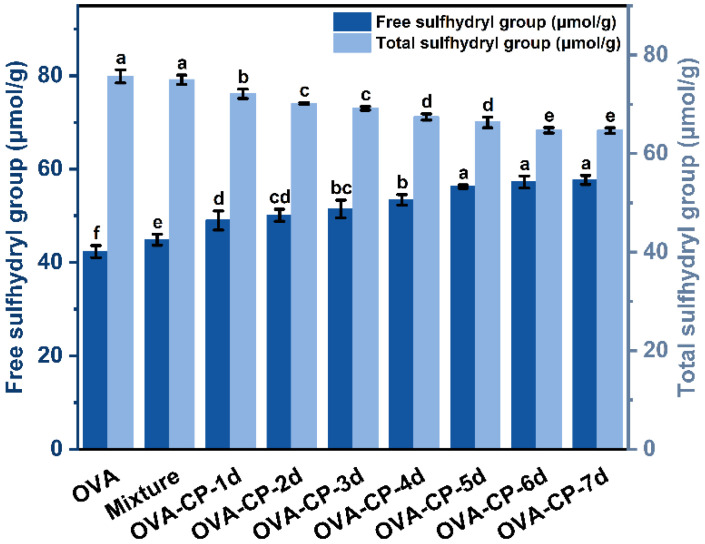
Free and total sulfhydryl content of native OVA, Mixture, and OVA–CP conjugates. Different letters indicate significant differences (*p* < 0.05).

Sulfhydryl groups (−SH) are critical for the structure and function of proteins, with their content closely related to protein denaturation and alterations in functional characteristics [38]. Free sulfhydryl groups refer to those that are soluble in the protein structure and not involved in disulfide bond formation, while total sulfhydryl content encompasses both free sulfhydryl groups and those engaged in disulfide bonds. This study analyzes the free and total sulfhydryl contents of various samples to elucidate the effects of glycosylation modifications on protein structure. Figure 6 illustrates the variations in free and total sulfhydryl content in OVA and its conjugates collected at different durations during the Maillard reaction. The results indicated distinct trends for free and total sulfhydryl content as the duration of the glycosylation reaction increased. The free sulfhydryl content of native OVA was measured at 42.32 μmol/g. This gradually increased with extended reaction duration, reaching a maximum of 57.66 μmol/g on day 7. This increase in free sulfhydryl content can be attributed to the unfolding of the protein structure or the cleavage of disulfide bonds during the glycosylation, which exposes previously concealed free sulfhydryl groups [39]. Conversely, total sulfhydryl content exhibited minimal overall changes, showing a slight decrease as the Maillard reaction progressed. This reduction may result from the oxidation of sulfhydryl groups under heating conditions, leading to diminished total sulfhydryl content. These findings align with previous research by Luo et al. [40], who reported a boost in free sulfhydryl content in giant salamander skin collagen during glycosylation modifications facilitated by ultrasonic treatment.

### 3.7. Particle Size and Polydispersity Index (PDI) Analysis

**Figure 7:**
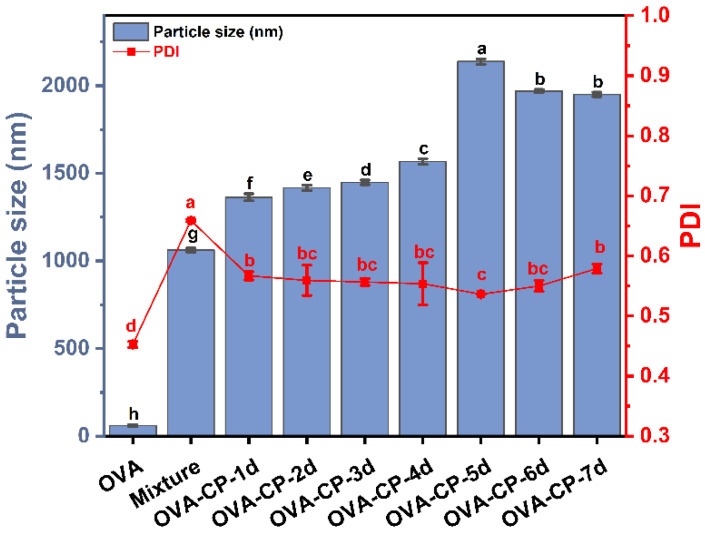
Particle size and polydispersity index (PDI) of native OVA, Mixture, and OVA–CP conjugates. Different letters indicate significant differences (*p* < 0.05).

Particle size and PDI are critical parameters for characterizing the dispersion state of proteins in aqueous solutions. The particle size reflects the aggregation or disaggregation state of proteins, while PDI measures the uniformity of protein dispersion, with a lower PDI reflecting a more uniform distribution and a higher PDI indicating less uniform dispersion [22]. These parameters help assess how glycosylation affects the molecular state and dispersion properties of proteins. The particle size and PDI of OVA and the OVA–CP conjugates were measured using a nanoparticle analyzer, as depicted in Figure 7. The particle size of native OVA was the smallest, measuring 60.14 nm. After mixing with CP, the particle size rapidly increased to 1062.33 nm (*p* < 0.05). As the Maillard reaction progressed, the particle size of the OVA–CP conjugates gradually increased, reaching a maximum of 2138.33 nm on day 5. This increase may be due to the gradual unfolding and aggregation of protein molecules during the glycosylation. Moreover, glycosylation may promote interactions between proteins and citrus pectin molecules, leading to the formation of larger aggregates and subsequently increased particle size. However, as the reaction progresses, the particle size of the conjugates begins to decrease on days 6 and 7, possibly due to the further breakdown of protein aggregates or the stabilization of molecular interactions. The PDI of OVA is 0.45, suggesting a uniform particle size distribution. Following mixing with CP, the PDI significantly increases to 0.66, reflecting a less uniform particle size distribution. After glycosylation modification, the PDI of the conjugates remains relatively stable, ranging from 0.4 to 0.6 throughout the reaction process. On day 5, the PDI of OVA–CP conjugates is recorded at 0.54, indicating a relatively uniform distribution of particle sizes.

### 3.8. Zeta Potential Analysis

**Figure 8:**
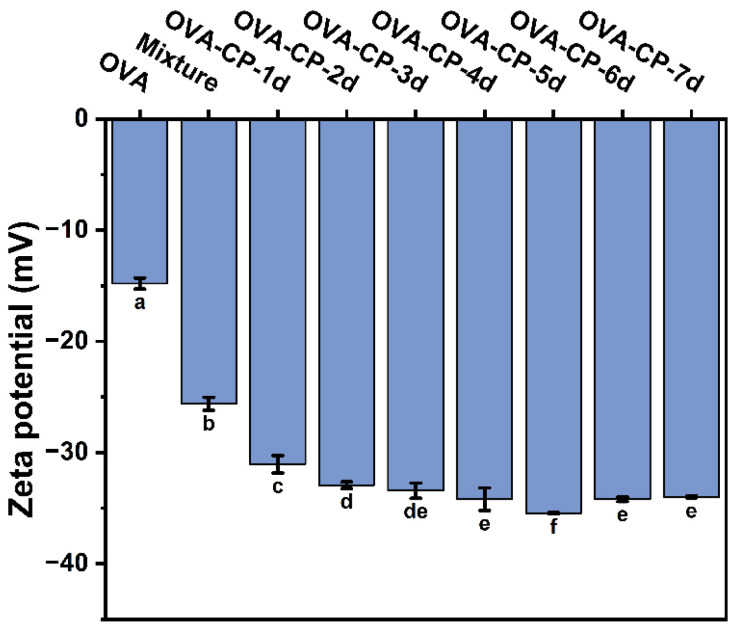
Zeta potential of native OVA, Mixture, and OVA–CP conjugates. Different letters indicate significant differences (*p* < 0.05).

Zeta potential is an important parameter that describes the charge state of protein molecules in solution, reflecting the intensity and sign of the surface charge [41]. The magnitude of the zeta potential correlates with the tendency of proteins to aggregate, precipitate, or repel each other. Generally, a higher absolute zeta potential indicates stronger electrostatic repulsion between proteins, thereby enhancing the stability of the system. As shown in Figure 8, all samples exhibited a negative zeta potential, with native OVA measuring −14.80 mV. After mixing with CP, the zeta potential of the Mixture increased to −25.63 mV, likely because CP is an anionic polysaccharide that increases the amount of negative charge on the protein surface [19]. Following glycosylation, the zeta potential further increases, reaching −35.47 mV on day 5. This substantial increase indicates a greater density of negative charges introduced during glycosylation, primarily from hydrophilic carboxyl and hydroxyl groups [42]. Additionally, glycosylation may alter the protein conformation, further exposing more negatively charged sites and enhancing the negative potential. These findings align with previous research [33].

### 3.9. Solubility Analysis

The solubility of proteins is closely related to their foaming properties, as proteins must dissolve in the aqueous phase to migrate to the air/water interface and form stable foams. Enhanced solubility facilitates the quick diffusion of proteins to the interface, where they can form a protective layer around bubbles, preventing coalescence and rupture [43]. Conversely, low solubility hinders effective migration to the air/water interface, resulting in reduced foaming capability and foaming stability. Therefore, increasing a protein’s solubility typically enhances its foaming properties and foam stability. The solubility of native OVA is 95.23%, which increases to 96.58% upon mixing with CP (Table 2). This enhancement in solubility can be attributed to the interaction between CP and OVA through weak electrostatic interactions or hydrogen bonding. With the progression of the Maillard reaction, the solubility of OVA–CP conjugates can increase to 98.95% (*p* < 0.05). Glycosylation enhances the hydrophilicity of the protein by introducing hydrophilic groups on its surface, thereby increasing its solubility in water [44]. Previous studies have reported similar improvements in solubility for soybean protein isolate and pea protein isolate following glycosylation [24,45]. However, after day 3, a slight decrease in the solubility of the OVA–CP conjugates was observed, although the solubility value remained above 95%, indicating good solubility. Overall, the Maillard reaction with CP significantly enhances the solubility of OVA, which likely contributes to the development of its foaming properties.

### 3.10. Foaming Properties Analysis

**Figure 9:**
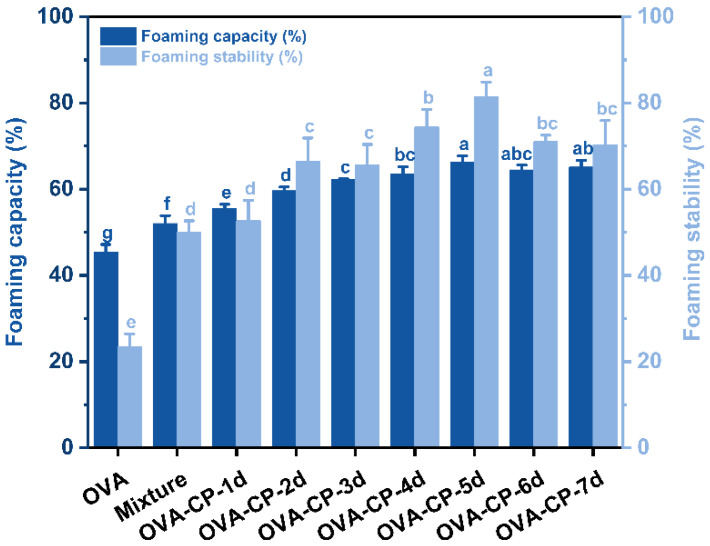
Foaming capacity and stability of native OVA, Mixture, and OVA–CP conjugates. Different letters indicate significant differences (*p* < 0.05).

Measuring the foaming capacity and stability of proteins is essential for evaluating their potential as foaming agents, particularly in food products like cakes and creams that require foaming and stable structures [46]. Foaming capacity reflects the ability of proteins to generate foams during whipping or mixing, while foam stability indicates their ability to maintain foam structure and resist collapse. As shown in Figure 9, there are notable differences in foaming capacity and stability between OVA and OVA–CP conjugates. Native OVA has a relatively low foaming capacity of approximately 45.33%, with a stability of 23.39%. However, mixing with citrus pectin notably improves both foaming capacity and stability (*p* < 0.05), which increase to about 52.01% and 50.02%, respectively. This improvement is likely due to the non-covalent interactions between citrus pectin and OVA at the air/water interface, which promote quick adsorption at the interface, enhancing the resistance of the system to foam rupture and collapse, and thereby enhancing the foaming properties of the Mixture [47]. At a Maillard reaction duration of 5 days, the foaming capacity of the OVA–CP conjugates reached an optimal level of 66.22%, with stability at 81.49%. Compared to natural OVA, the foaming capacity and stability of the OVA–CP conjugates formed after 5 days of the Maillard reaction increased by 46.09% and 248.37%, respectively. This may be attributed to a denser interfacial layer, which forms a complex cross-linked network around the bubbles [48]. Additionally, a higher zeta negative potential effectively prevents foam coalescence and aggregation. Furthermore, the covalently bonded polysaccharide chains of citrus pectin extend into the aqueous phase, creating a more viscoelastic interfacial layer with steric hindrance, thus enhancing the foam stability of the conjugates [49]. However, when the reaction duration exceeds 5 days, further increases in foaming capacity are not observed, and foam stability slightly decreases. This suggests that excessive Maillard reaction conditions may negatively impact the foaming performance of the proteins.

**Figure 10:**
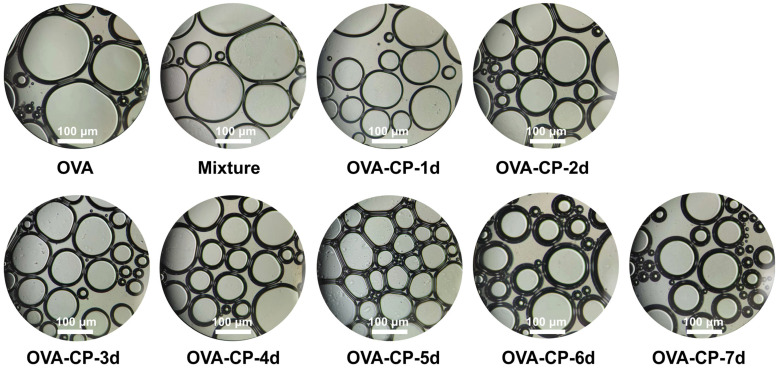
Micrographs of various foam samples of native OVA, Mixture, and OVA–CP conjugates after 30 min.

Optical microscopy was utilized to observe the microstructure of foams, aiming to understand the morphology, size distribution, and arrangement of bubbles within the foam. Microscopic observations provide visual insights into bubble size, shape, and the thickness of the interfacial layer, further elucidating the effects of different degrees of the Maillard reaction on foam formation and stability. Figure 10 displays the microstructure of various foam samples after 30 min. The results indicate that natural OVA produces larger, unevenly distributed bubbles with a thin interfacial layer. In contrast, foams created from the OVA–CP conjugates showed a significantly greater quantity of smaller bubbles with a more uniform size distribution. This suggests that the Maillard reaction enhances the foaming capacity and stability of OVA, with minimal aggregation or dispersion phenomena. The addition of citrus pectin facilitates the formation of a more elastic interfacial film at the air/water interface, effectively preventing the expansion and coalescence of foams [50,51]. Additionally, glycosylation modification effectively reduces foam size and promotes a denser arrangement, enhancing foam stability. Meng et al. [52] demonstrated that treatment with naringin improved the interfacial and foaming properties of chickpea protein. Overall, glycosylation modification based on the Maillard reaction significantly improves the foaming properties of OVA.

## 4. Conclusions

This study successfully prepared OVA–CP conjugates through the Maillard reaction and evaluated the impact of glycosylation modifications on the structure and foaming properties of OVA. The findings indicated that the degrees of both grafting and browning confirm the occurrence of the Maillard reaction between OVA and CP, with peak grafting observed on day 5. SDS-PAGE analysis substantiated the formation of high-molecular-weight conjugates through covalent bonding, which was further validated by Fourier-transform infrared spectroscopy. Moreover, glycosylation modifications significantly altered the secondary and tertiary structures of OVA, leading to increased surface hydrophobicity. The analysis of foaming properties and microstructure revealed that glycosylation significantly improved the foaming capacity and stability of OVA. Particularly, the foam generated from the OVA–CP conjugates on the fifth day exhibited smaller bubble sizes and denser interfacial layers, which effectively reduced bubble coalescence and aggregation. In summary, moderate glycosylation modifications substantially enhance the foaming capacity and stability of OVA. OVA–CP conjugates hold significant potential for applications in food products that require stable foam structures, serving as natural stabilizers and foaming agents. However, obtaining OVA–CP conjugates with optimal foaming properties requires a significant time commitment (5 days), which may hinder their potential for industrial applications. Future research should not only focus on the adsorption kinetics and interfacial dilational rheology of OVA and OVA–CP conjugates at the air/water interface, but also on their application in milk beverages and baked goods to better understand their functionality and suitability in real food systems.

## Figures and Tables

**Table 1:** Secondary structure content of native OVA, Mixture, and OVA–CP conjugates.

Sample	α-Helix (%)	β-Sheet (%)	β-Turn (%)	Random Coil (%)
OVA	12.20	35.33	40.34	12.13
Mixture	22.77	33.51	33.03	10.69
OVA–CP–1d	22.51	34.27	32.53	10.68
OVA–CP–2d	22.69	33.82	33.05	10.46
OVA–CP–3d	20.38	36.96	31.07	11.58
OVA–CP–4d	22.26	36.02	31.12	10.60
OVA–CP–5d	22.02	37.14	30.14	10.70
OVA–CP–6d	21.84	36.17	31.42	10.57
OVA–CP–7d	21.78	38.78	28.65	10.79

All data are expressed as their mean ± standard deviation.

**Table 2:** Solubility of native OVA, Mixture, and OVA–CP conjugates.

Sample	Solubility (%)
OVA	95.23 ± 0.38 d
Mixture	96.58 ± 0.11 c
OVA–CP–1d	97.29 ± 0.28 bc
OVA–CP–2d	97.72 ± 0.23 b
OVA–CP–3d	98.95 ± 0.15 a
OVA–CP–4d	98.02 ± 0.73 b
OVA–CP–5d	94.92 ± 0.37 ad
OVA–CP–6d	93.60 ± 0.90 e
OVA–CP–7d	94.44 ± 1.06 de

All data are expressed as mean ± standard deviation; different lowercase letters represent significant (*p* < 0.05) differences between groups in the same column.

## Data Availability

The original contributions presented in the study are included in the article, further inquiries can be directed to the corresponding author.
